# Whole genome sequencing and analysis of fenvalerate degrading bacteria *Citrobacter freundii* CD-9

**DOI:** 10.1186/s13568-022-01392-z

**Published:** 2022-05-06

**Authors:** Xuerui Zhou, Dan Lei, Jie Tang, Min Wu, Hong Ye, Qing Zhang

**Affiliations:** Key Laboratory of Food Biotechnology, School of Food and Biotechnology, Xihua University, Chengdu, 610039 Sichuan People’s Republic of China

**Keywords:** Pyrethroids, Bioremediation, Genomics, RT-qPCR

## Abstract

*Citrobacter freundii* CD-9 is a Gram-negative bacteria sourced from factory sludge that can use fenvalerate as its sole carbon source and has a broad degradation spectrum for pyrethroid pesticides. The whole genome of CD-9 sequenced using Illumina HiSeq PE150 was reported in this study. The CD-9 genome size was 5.33 Mb and the G + C content was 51.55%. A total of 5291 coding genes, 9 5s-rRNA, and 79 tRNA were predicted bioinformatically. 3586 genes annotated to the Kyoto Encyclopedia of Genes and Genomes (KEGG) database that can be involved in 173 metabolic pathways, including various microbial metabolic pathways that degrade exogenous chemicals, especially those that degrade aromatic compounds, and also produce a variety of bioactive substances. Fifty genes related to pyrethroid degradation were identified in the *C. freundii* CD-9 genome, including 9 dioxygenase, 25 hydrolase, and 16 esterase genes. Notably, RT-qPCR results showed that from the predicted 13 genes related to fenvalerate degradation, the expression of six genes, including esterase, HAD family hydrolase, lipolytic enzyme, and gentisic acid dioxygenase, was induced in the presence of fenvalerate. In this study, the key genes and degradation mechanism of *C. freundii* CD-9 were analyzed and the results provide scientific evidence to support its application in environmental bioremediation. It can establish application models for different environmental pollution management by constructing genetically engineered bacteria for efficient fenvalerate or developing enzyme formulations that can be industrially produced.

## Introduction

Pyrethroids (PYRs), which are considered a safe alternative to organophosphorus pesticides, are a class of highly effective and low-toxicity biomimetic insecticides that are universally used to improve agricultural productivity and act as the second largest group of pesticides following only organophosphorus formulations (Katherine et al. 2012). PYRs are classified into a dual classification of Type I and Type II based on their mechanism of toxicity and chemical structure (Bardullas et al. 2015). Fenvalerate, a Type II PYR, is among the most commonly used pyrethroid insecticides for controlling pest insects afflicting crops such as cotton and vegetables (Zhang et al. 2018). Following massive use of fenvalerate, it was detected in sediment, soil, and rivers (Mulla et al. 2017) and has become a chemically harmful substance that threatens human safety and destroys the integrity of ecosystems. Fenvalerate is highly hydrophobic, strongly absorptive, and cause toxic side effects in humans, such as reproductive toxicity (Fei et al. 2010), genotoxicity (Wang et al. 2017) and neurotoxicity (Gu et al. 2010). Therefore, it is imperative to eliminate or degrade pyrethroid pollutants within the environment. Microbial remediation is an environmentally and economically friendly approach that does not damage natural processes when compared with other degradation methods (Zhan et al. 2018a, b). In one study, 12 bacterial strains (*Curtobacterium* sp.*, Kocuria* sp*.* and *Lysinibacillus* sp.) were isolated from Brazilian savannah, sea, and the tropical peat normally described as “turfa” soil to accelerate the degradation of esfenvalerate and a biometabolic pathway was proposed (Birolli et al. 2016). In a separate study, a strain of *Bacillus licheniformis* CY-012 was isolated from soil in a garden that had been sprayed with pyrethroids, and the bacteria was found to efficiently degrade 80% fenvalerate approximately (Tang et al. 2018). *Citrobacter* spp. are part of the normal flora of human and animal intestines and have been reported to degrade not only phenol, tannic acid, and lignocellulose, but also to reduce the toxicity of methyl parathion and *p*-nitrophenol under certain conditions (Deng et al. 2018; Kumar et al. 1999; Pino and Peñuela 2011; Wang et al. 2021). *Citrobacter* spp. isolated from farmland soil can effectively degrade four types of organophosphorus pesticides, with a degradation rate between 58 and 96% (Jiang et al. 2019). However, there have been few studies on the application of *Citrobacter* spp. for the degradation of pyrethroids.

To date, research on the microbial degradation of pyrethroids has mainly concentrated on the screening and identification, degradation characteristics, and degradation pathways. However, only a few studies have been carried out on microbially produced degradative enzymes and genes. The key degradation enzymes (oxygenases, carboxylesterases, and aminopeptidases) in the microbial degradation of pyrethroids have been purified and characterized (Chen et al. 2013; Cai et al. 2017; Tang et al. 2017). By isolating and characterizing these functional enzymes, detailed mechanisms of microbial degradation can be determined and pyrethroid-contaminated environments can be bioremediated (Hu et al. 2019). Recently, with the rapid development of genetic engineering and other technologies, cloning and expression of degrading enzyme genes has made great progress. The carboxylesterase EstSt7 from *Sulfolobus tokadaii,* has been shown to hydrolyze a variety of synthetic pyrethroids, including Type I and Type II pyrethroid pesticides (Wei et al. 2013). The pyrethroid-degrading gene coding for Est3385 from *R. palustris* PSB-S was also cloned and expressed using previously published genes (Luo et al. 2018). The construction of pesticide-degrading engineered bacteria with broad-spectrum and efficient degradation ability by mining more functional genes has attracted much attention, and will open new avenues for the remediation of environmental pollution.

The mining of key degradation genes at the sequences level is achieved mainly through the whole genome sequencing of microbial strains and in depth bioinformatic analyses. Therefore, the analysis of the degradation mechanism at the gene level has become a breakthrough. *Burkholderia* sp. CQQ001 (B. CQ001) which could degrade dexamethasone effectively was sequenced using Illumina Hiseq4000 in combination with third-generation sequencing technology to analyze the functional genes and metabolic pathways involved in dexamethasone degradation (Si et al. 2019). The whole genome of *Bacillus tropicus* strain AOA-CPS1 (BtAOA) was sequenced using the Pacific Biosciences RS II sequencer to reveal that the strain harbored genes intimately related to the biodegradation of numerous chlorophenolic compounds (Aregbesola et al. 2021). As far as is known, only small number of studies have focused on the mechanism of pyrethroid degradation by analysis of whole genome sequences. One such study deciphered the complete genomic sequence information of deltamethrin-degrading bacterium ZJ6, which was then used to provide a theoretical basis for further elucidation of its degradation properties (Hao et al. 2018).

In this study, to explore the genes related to the degradation of fenvalerate compounds, whole genome sequencing analysis of a recently reported strain of *Citrobacter freundii* called CD-9, isolated from plant sludge, that degraded 88% of fenvalerate at pH 6.3, inoculum of 6% (v/v), and substrate concentration of 77 mg L^−1^ (Tang et al. 2020), was carried out on the Illumina Solexa sequencing platform. The predicted genes were annotated using databases including Gene Ontology (GO), Clusters of Orthologous Groups (COGs) proteins, and the Kyoto Encyclopedia of Genes and Genomes (KEGG). The expression of putative functional genes was experimentally verified using RT-qPCR. These results provide more favorable support for further exploration of the degradation mechanisms of pyrethroids by *C. freundii* CD-9, construction of genetically engineered pyrethroid-degrading bacteria, and heterologous expression of enzyme preparations that can be industrially produced.

## Materials and methods

### Bacterial, growth conditions, and chemicals

*Citrobacter freundii* CD-9 was isolated by enrichment culture from the sludge of a sewage pipeline at Pesticide Chemical Co., Ltd., Chengdu Province, China. This strain was preserved at the China General Microbiological Culture Collection Center (Collection number: CGMCC 20106). CD-9 was cultured in Luria-Bertani (LB) culture medium (5 g L^−1^ yeast extract, 10 g L^−1^ tryptone, and 10 g L^−1^ NaCl) and LB agar plates (5 g L^−1^ yeast extract, 10 g L^−1^ tryptone, 10 g L^−1^ NaCl, and 20 g L^−1^ agar powder) at 30 °C overnight.

Pesticides fenvalerate, deltamethrin, fenpropathrin, bifenthrin, and beta-cypermethrin (purity 96%), were purchased from Nanjing Rongcheng Chemical Company, China. Chromatography-grade acetonitrile was purchased from Shanghai Titan Scientific Co., Ltd. Pyrethroids were dissolved in acetonitrile to a stock concentration of 10 g L^−1^ and diluted to the desired concentration in culture medium. Single colonies were selected after 24 h of incubation and cultured in liquid LB medium at 30 °C with shaking (180 rpm) until the cultures reached the logarithmic phase.

### Broad-spectrum analysis of CD-9 supernatants

The extraction of residual fenvalerate, deltamethrin, fenpropathrin, bifenthrin, and beta-cypermethrin from the media was performed pursuant to the method depicted by Tang et al. (2013). Equal volumes of acetonitrile were added to well mixed cultures, followed by ultrasonic-assisted extraction for 30 min and centrifugation at 13,000×*g* for 10 min. The supernatant was collected, while filtered through a 0.45-μm organic filter membrane, and the residual concentration of pyrethroids were measured by using high-performance liquid chromatography (HPLC).

### Genome sequencing, assembly, and annotation of CD-9

*Citrobacter freundii* CD-9 was cultured overnight in LB culture medium at 30 °C for 48 h. Bacterial cells were collected by centrifugation, and a bacterial genomic DNA extraction reagent kit (Shanghai Sangon Biotech Co., Ltd.) was subsequently used to extract the genomic DNA. Next, 1% agarose gel electrophoresis and micro-spectrophotometry were used to determine the quality and concentration of the extracted DNA solution. Sequencing was performed by using an Illumina HiSeq PE150 second-generation sequencer, and Trimmomatic 0.36 was taken to handle to obtain high-quality clean reads to cast aside adapter sequences, low-quality bases, and ambiguous reads (Yadav and Dubey, 2018). High-quality reads were used for de novo assembly using the SPAdes 3.5.0 genome assembler (Bankevich et al. 2012). The GapFiller, version 1.11 was used to fill with gap in the scaffold, and the sequence data were corrected by PrInSeS-G 1.0.0 to fix editing errors and the missing insertion of small fragments during the splicing process (Xu et al. 2020).

The coding genes, rRNAs, and tRNAs were predicted using Prodigal, RNAmmer, and Aragorn, respectively (Lagesen et al. 2007). Moreover, the assembly results of the gene components were predicted using Rapid prokaryotic genome annotation (reProkka) 1.10 software (Xu et al. 2020). The protein-coding genes were annotated using multiple databases at NCBI by BLAST+ analysis, including euKaryotic Ortholog Groups (KOG), Clusters of Orthologous Groups of proteins (COG), Non-Redundant Protein Sequences (NR), Nucleotide Sequences (NT), PFAM, Swiss-Prot, and TrEMBL. Gene Ontology (GO) annotation was performed by comparing the gene sequence with the Swiss-Prot and TrEMBL databases (Ashburner et al. 2000). KEGG annotation was performed using the KAAS 2.1 server (Ogata et al. 1999). The whole-genome sequences of CD-9 were deposited in the NCBI database and the accession number is JAKQYL000000000.

### Prediction of fenvalerate degradation genes

Related functional genes of degrading enzymes were summarized according to the functional annotations of CD-9 coding genes and combined with the reported functional annotation results of pyrethroid-degrading enzyme genes. The reported pyrethroid degradation genes used as reference genes were firstly matched for homology with the functional genes annotated in the genome, and then 13 genes with high homology were initially predicted for validation. The neighbor-joining method was taken to construct phylogenetic trees with 1000 replicates in MEGA 6.0 software (Tamura et al. 2013).

### Synthesis of primers

According to the coding sequence (CDS) of the predicted genes, the primer design software Primer version 5 was used to design primers for amplification of each fragment of each predicted gene as well as a 16S rRNA fragment, avoiding primer dimers, hairpin structures, mismatches, etc. (Table 1), and the selected primer sequences were sent to Chengdu Qingke Technology Co., Ltd. for synthesis.

**Table 1:** Primers for amplification of predicted and 16S rRNA genes

Target fragments	Forward primer sequence (5ʹ → 3ʹ)	Reverse primer sequence (5ʹ → 3ʹ)	Function annotation
*EstP*1	GTGAAGCCGTGGCGTTGT	GTGCGGTTTGCTGTAGGG	Beta-phosphoglucomutase
*EstP*2	ACGCCACAAACCTGCTCC	CGCCGCCTGTATTCCAAA	Beta-phosphoglucomutase
*EstP*3	TCCAGGCGTTTGGCATTA	CGACACCGTGCTCTTCGT	Beta-phosphoglucomutase
*EstP*4	ATCAGGTCGGCGAGTATTT	GCGGTTATCCCAGTTGTT	Glucose-6-phosphate 1-dehydrogenase
*EstP*5	AGCCTTACGGTACGACTTCC	ACGCCGCTTTGTTTCTGC	Putative hydrolase
*EstP*6	TATGGCTACAACCGTCCTG	ATCACCGTGGATCACCAG	Pimeloyl- [acyl-carrier protein] methyl ester esterase
*EstP*7	GAGGCGTTGTTTGAGGCA	CAGGGTCTGGGCTTTGATT	2-Hydroxy-6-ketonona-2,4-dienedioic acid hydrolase
*EstP*8	CTGGCGACTATTCCCTCAA	CGTAAAGCCCATACCACAAC	Monoterpene epsilon-lactone hydrolase
*EstP*9	GTCGATCTGCGAGAACCG	CCGCCTTAGCAAACACCA	Pimeloyl- [acyl-carrier protein] methyl ester esterase
*EstP*10	GCGTGCTGGTGCTGGAAA	GACTGGTTGTGGCGGTGA	Gentisate 1,2-dioxygenase
*EstP*11	TTCTGTTTAGGCGTGGGAGC	CATGTTGTAGGACACGGCAAG	2,3-Dihydroxyphenylpropionate 1,2-dioxygenase
*EstP*12	CTGCTGGAATTTCCGTTTG	GCATTGCCCATTGCTGTT	4-Carboxymuconolactone decarboxylase
*EstP*13	GTCGGCATTTATCGTATGG	GTGATGGCAATCGGAAGA	4-Hydroxybenzoate decarboxylase
*16S rRNA*	CGCTACCATGCAGTCGAACG	GGTCCCCCTCTTTGGTCTTG	–

### RNA extraction and reverse transcription polymerase chain reaction (RT-qPCR)

Bacteria cultured to the logarithmic growth phase were collected via centrifugation at 12,000×*g* at 4 °C for 2 min. Total RNA was extracted as stated by the operating steps of the E.Z.N.A.^®^ Bacterial RNA Kit and stored at − 80 °C. The integrity of RNA was verified via 1% agarose gel electrophoresis, and the determination of RNA concentration and purity were used a micro-spectrophotometer.

The TB Green^®^ Premix Ex TaqTM II (Tli RNaseH Plus) manual was used to prepare the reaction solutions. First, 7 μL RNA was used for the genomic DNA removal reaction, and then 10 µL of the reaction solution was used as a template for reverse transcription as follows: PrimeScript RT Enzyme Mix I 1 μL, RT Prime Mix*4 1 μL, 5× PrimeScript Buffer 2 (for Real Time) 4 μL, and RNase-free water to make up to 20 μL. Reactions were incubated at 42 °C for 15 min then the enzyme was inactivated at 85 °C for 5 s. cDNA was stored at − 20 °C.

The operating method of Applied Biosystems StepOnePlus™ System (ABI, Carlsbad, USA) was used with TB Green™ Premix Ex Taq™ (Takara, Tokyo, Japan). DNA (2 µL) from the experimental group and the control group was used as a template for RT-qPCR. There were six parallels for each sample, and the average value was then taken. The total volume of RT-qPCR reactions was 20 μL, with each containing 10 μL TB Green Premix Ex Taq II, 0.8 μL ach of PCR forward and reverse primers, 6 μL ddH_2_O, and 0.4 μL ROX Reference Dye (50×). The reaction conditions were as follows: 95 °C for 30 s, 40 cycles at 95 °C for 5 s, and 60 °C for 30 s. A Qtower 2.0 real-time fluorescent quantitative PCR instrument was used to automatically construct amplification curves, standard curves, and melting curves. The relative expression level was reckoned as the fold change in accordance with the 2^−△△Ct^ method (Livak and Schmittgen 2001) according to the following equation:

$$ \Delta {\text{CT}}_{{({\text{sample}})}} = {\text{CT}}_{{({\text{target gene of sample}})}} - {\text{CT}}_{{({\text{16S rRNA of sample}})}} . $$

$$ \Delta {\text{CT}}_{{({\text{control}})}} = {\text{CT}}_{{({\text{target gene of control}})}} - {\text{CT}}_{{({\text{16S rRNA of control}})}} . $$

$$ {\text{Relative expression}} = {2}^{{ - (\Delta {\text{CT }}\left( {{\text{sample}}} \right){-}\Delta {\text{CT }}({\text{control}}))}} $$

### HPLC conditions and analysis

The concentration of pyrethroids was determined using a Waters 2695 HPLC (Waters, Milford, MA, USA) equipped with a ZORBAX eclipse plus C18 column (4.6 mm × 150 mm × 5 µm). The pyrethroids (10 g L^−1^) were diluted in acetonitrile to prepare standard solutions with a concentration of 50 mg L^−1^. Pyrethroids were identified and quantified depended on the retention time and peak area of the pure standard. The degradation rate was calculated using the following equation:

$$ {\text{The degradation rate }}\left( \% \right) = \left( {{1} - {\text{C}}/{\text{C}}_{0} } \right) \times {1}00 $$

where *C* and *C*_0_ represent the pyrethroids concentrations in the inoculated and non-inoculated media, respectively.

## Results

### Substrate spectrum analysis of CD-9

Peak area-mass linear fitting was performed with the same concentrations of different pyrethroid pesticide standard solutions, and the respective standard curve equations were obtained, as shown in Table 2. In the standard curve equation, y is the HPLC peak area of the pesticide (mAU*s) and x is the mass of the pesticide (μg).

**Table 2:** Standard curve equation of pyrethroids

Pyrethroids	Standard curve equation	R^2^	Retention time (min)
Permethrin	y = 336,162 x + 1187	0.9998	6.428
Deltamethrin	y = 101,596 x − 1010	0.9993	10.547
Fenpropathrin	y = 284,100 x + 2471	0.9971	8.469
Bifenthrin	y = 482,670 x + 6405.6	0.9995	13.452
Beta-cypermethrin	y = 214,369 x − 4617	0.9988	10.147, 10.289

**Fig. 1:**
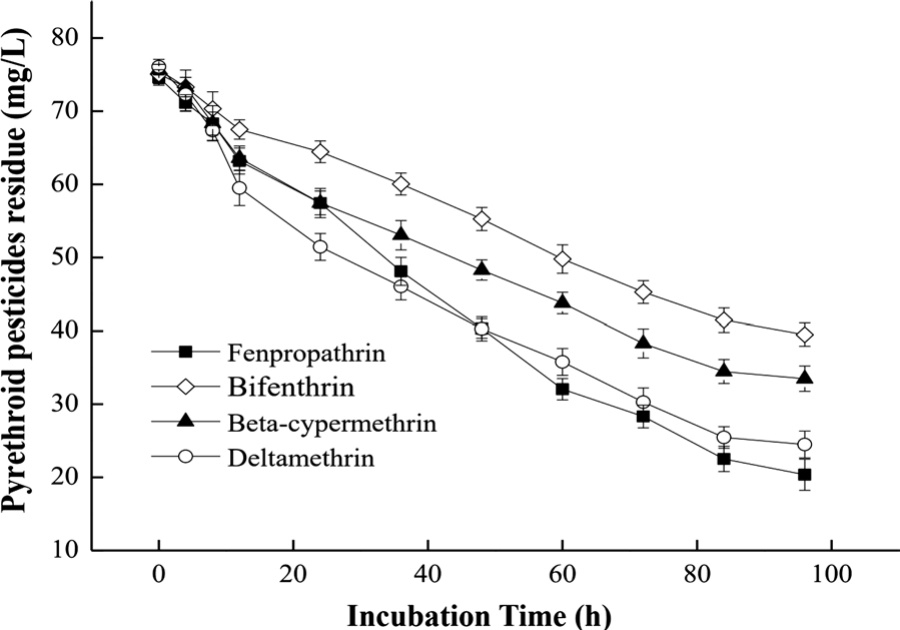
*C. freundii* CD-9 degradation of pyrethroids

The degradation rates of different pyrethroids when incubated with *C. freundii* CD-9 are shown in Fig. 1. It could be seen that the strains had a broad degradation spectrum for pyrethroids, and the degradation ability of pyrethroids with different structures was obviously different. Beta-cypermethrin had the fastest degradation rate, followed by that of deltamethrin and bifenthrin. The degradation amount of bifenthrin was slightly higher than for fenpropathrin and permethrin following 90 min of incubation. Different substituents or isomer forms in pesticide molecules may affect the related degrading enzymes secreted by strains, which may lead to differences in the degradation rate of similar pesticides. It has also been confirmed that bacterial cells are affected by the structure of many pollutants and the physical and chemical properties of the soil matrix (Zhang et al. 2020). It could be inferred that the broad metabolic spectrum of strain CD-9 toward different types of pyrethroids indicates that it has a complex metabolic capacity.

### Whole genome analysis of CD-9

**Fig. 2:**
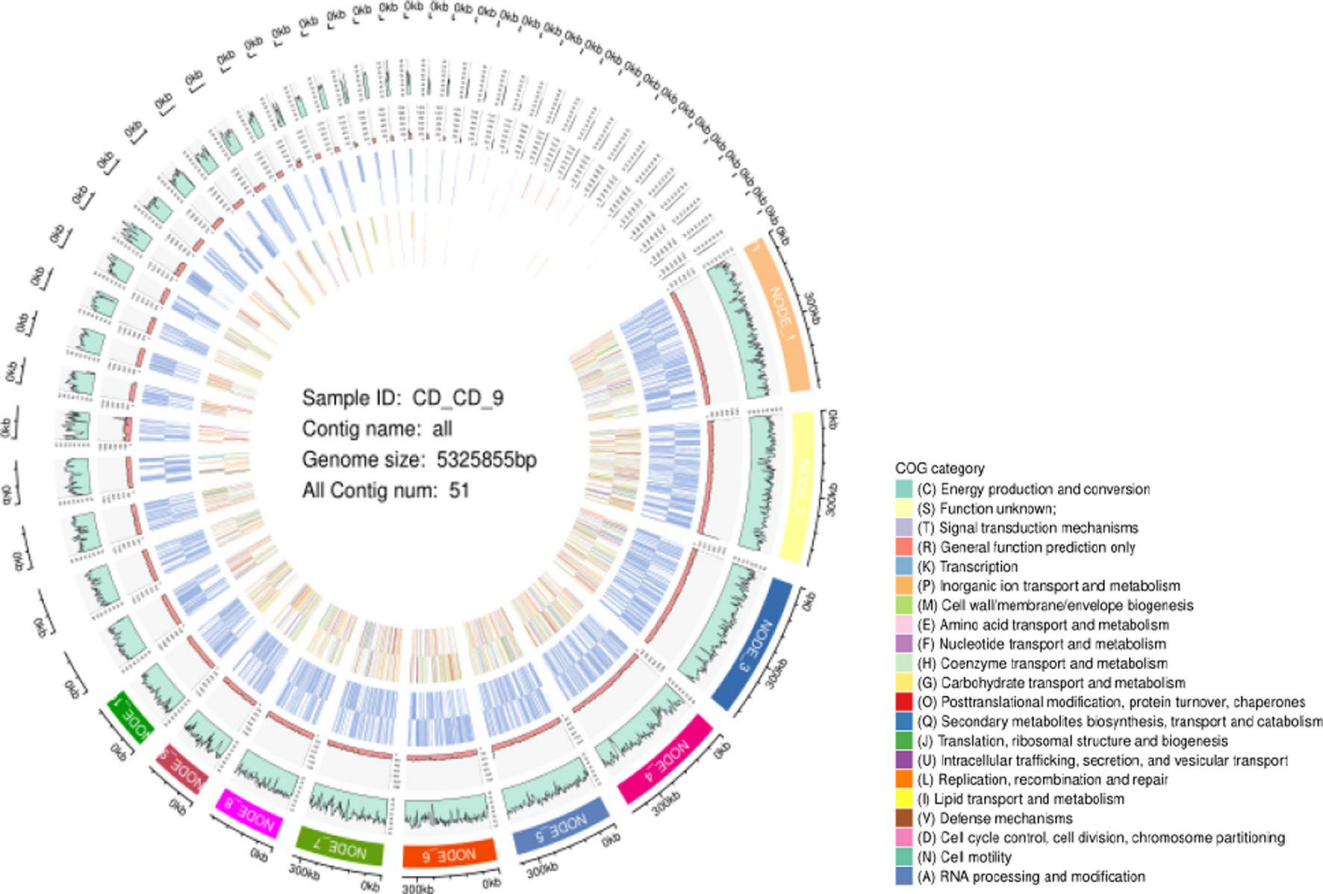
Draft genome *C. freundii* CGMCC 20106. The first circle is contigs and corresponding length information, the second circle is GC content information, the third circle is the corresponding base sequencing depth, the fourth circle and the fifth circle are CDS, rRNA, tRNA distribution information, and the sixth circle and the seventh circle are the corresponding COG function classification. (From outmost to innermost)

Genomic information helps to generate insights into the mechanisms of pyrethroid degradation by bacteria. Hence, we carried out whole-genome sequencing using Illumina Hiseq PE150 to decipher the complete set of genes involved in the degradation of pyrethroids. A total of 5291 coding genes, ranging in length from 38 to 11,199 bp with an average sequence length of 894.51 bp, were predicted in the genome of CD-9. The calculation method of (GC)/(G + C) was used to perform GC skew analysis and produce a schematic representation of the draft genome of CD-9, as detailed in Fig. 2. It has become clear from the results that the genome size was approximately 5.326 Mb, with a G + C content of 51.55% (Table 3). Good deal of genes predicted in this genome was analogous to that reported in numerous of the genomes of *Citrobacter* at NCBI (https://www.ncbi.nlm.nih.gov/).

**Table 3:** Molecular characteristics of the genome of *C. freundii* CD-9

Characteristics	*C. freundii* CD-9
Length (bp)	5,325,855
GC content	51.55
Coding ratio (%)	88.87
Total bases (bp)	4,732,830
Length variation (range in bp)	38–11,199
Average length (bp)	894.51
Repeat ratio (%)	0
Repeat region count	0
No. of ribosomal RNA	9
No. of transfer RNA	79

**Fig. 3:**
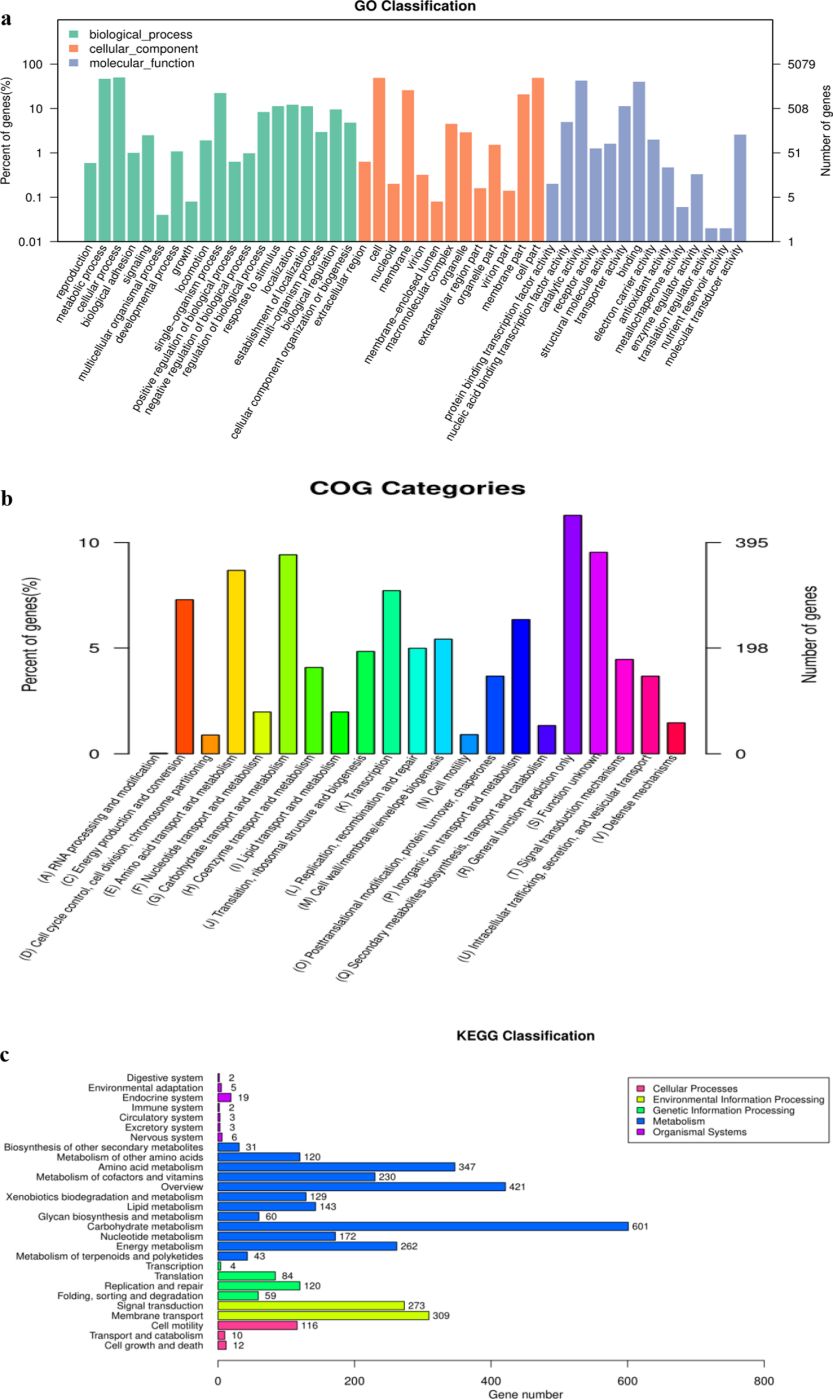
**a** The map of GOclassification annotation. **b** The map of COG classification annotation. **c** KEGG pathway classification histogram

The gene functional annotation consisted of COG, GO, KEGG, NR, Swiss-Prot, TrEMBL, and NT were got by aligning the coding genes in the *C. freundii* CD-9 genome using the BLAST database. Among the general function annotation results, GO, KEGG, and COG database annotations were more useful, accounting for 79.35, 44.87, and 77.77% genes, respectively. The NR database annotated 5057 coding genes in the genome of strain CD-9, while gene ontology (GO) annotated 4030 genes as 46 GO terms. As shown in Fig. 3a, the supreme number of functional genes was predicted in the biological process domain, followed by the molecular function domain, in which the number of genes related to catalytic activity was the largest, while no more than 50 genes were predicted in some GO terms.

The COG database was constructed by integrating the protein sequences of species that have been sequenced to date (Galperin et al. 2015). By analysis, the protein sequence can assign to a specific COG, and each COG cluster is inclusive of orthogonal sequences with the purpose that the function of the specific sequence can be deduced (Hao et al. 2018). The COG database was annotated to 3950 genes which were assigned to 26 functional groups. As shown in Fig. 3b, among the groups, a large number of genes involved in general function prediction were the most abundant, followed by proteins involved in carbohydrate transport and metabolism (372). Most of all, the number of genes involved in amino acid transport and metabolism was relatively high (n = 343). In addition, there were 305 genes involved in transcription and 288 genes associated with energy generation and transformation. In contrast, the lowest number of genes were annotated in the categories of RNA processing and modification (1) and cell motility (19).

### Analysis of degradation characteristics in genome of CD-9

To better comprehend the metabolic pathways of CD-9, we analyzed the genes identified by the KEGG database. A total of 3586 of the 5291 genes were annotated into 28 biological pathways in the KEGG database, of which 601 genes were assorted as carbohydrate metabolism genes, 347 genes were assorted as amino acid metabolism genes, and 309 genes were assorted as membrane transport genes (Fig. 3c).

**Fig. 4:**
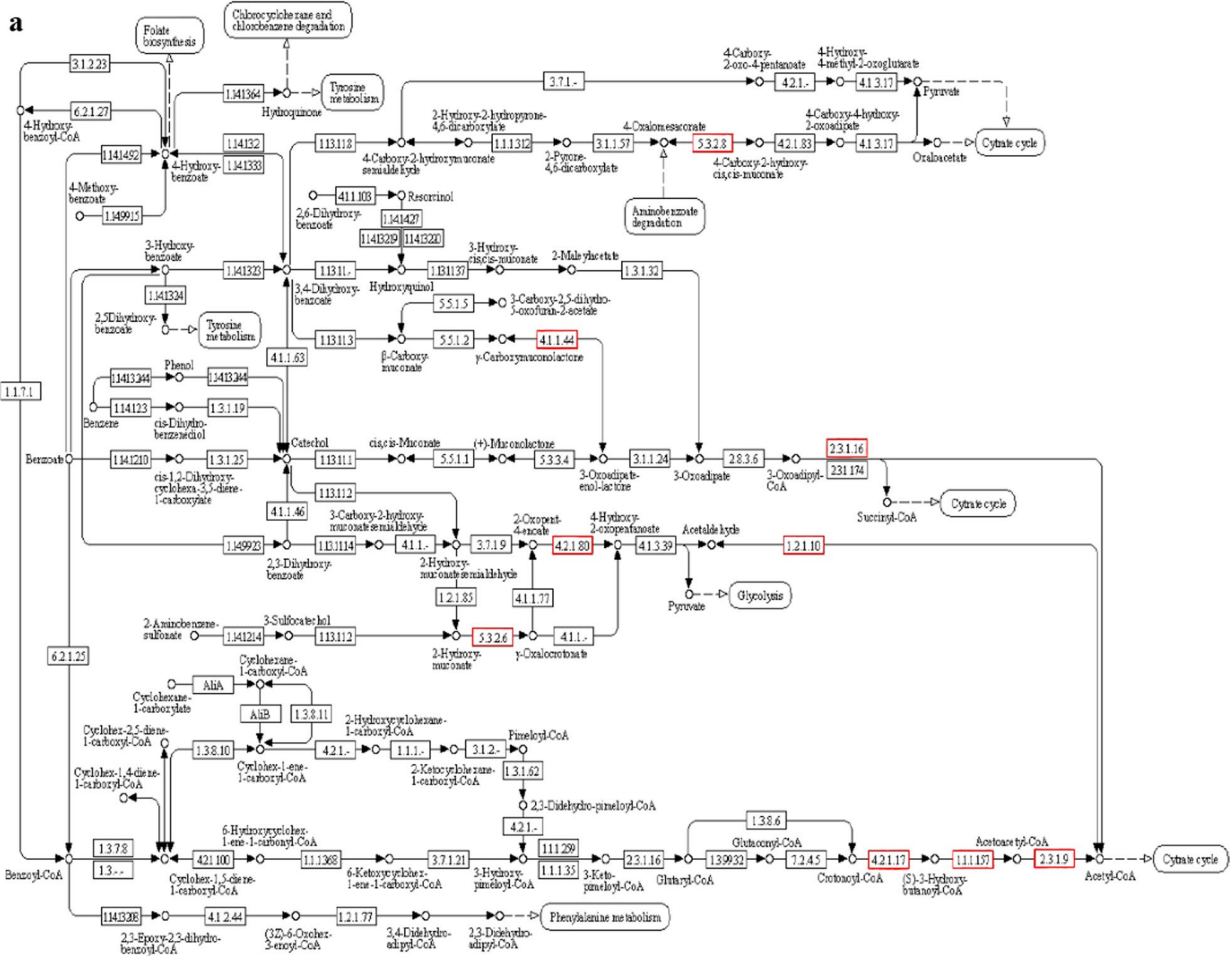

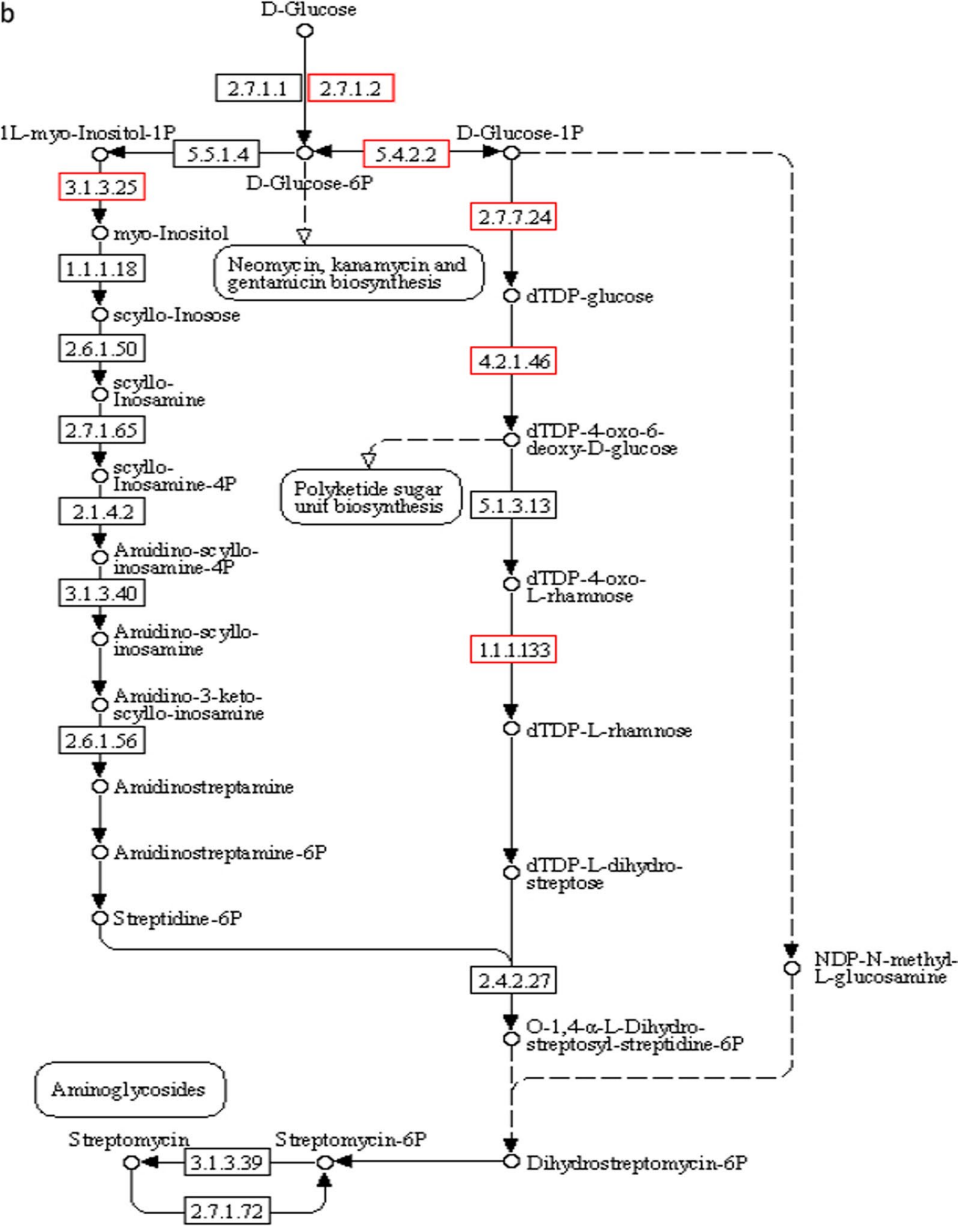
**a** Degradation pathway of benzoate. **b** Biosynthetic pathway of streptomycin. The red boxes represent the key genes contained in strain CD-9

Genes clearly associated with the metabolism of substances account for 70%, encompassing most of the microbial metabolic pathways and the catabolic pathways of secondary metabolites. In particular, the degradation pathways of aromatic compounds, such as aminobenzoate metabolism, naphthalene degradation, and toluene degradation, indicate that strain CD-9 has a good potential to degrade aromatic substances. Among these, it was analyzed that 17 genes were associated with benzoate in which, EC.2.3.1.9 (acetyl-CoA C-acetyltransferase), EC.1.1.1.157 (3-hydroxybutyryl-CoA dehydrogenase), and EC.5.3.2.6 (4-oxalocrotonate tautomerase), were found to take a crucial role in benzoate degradation (Fig. 4a). Furthermore, there were 129 genes annotated with exogenous metabolism in the CD-9 genome, among which six were concerned with the degradation pathway of atrazine, nine were concerned with the degradation pathway of dioxins, and ten were concerned with the degradation pathway of chlorinated alkanes, chlorinated alkenes, chlorinated cyclic hydrocarbons, and chlorobenzenes, indicating that strain CD-9 has the potential to metabolize other types of pesticides and halides simultaneously.

In addition, pathways for the synthesis of secondary metabolites were broadly distributed in the CD-9 genome as determined by KEGG database analysis, including streptomycin biosynthesis (Fig. 4b), tetracycline biosynthesis, pantothenate and COA biosynthesis, and phenylpropanoid biosynthesis. This suggests that the strain CD-9 has a good potential to degrade aromatic substances and can produce a variety of biologically active substances, such as antibiotics and other drugs, indicating that it is possible to identify biologically active substances from such strains to develop new drugs.

Above all, there are many unknown functional genes in CD-9 that are worthy of further mining and analysis, and they have great potential for application in crop production safety and ecological environment restoration.

### Prediction and screening of pyrethroid-degrading related genes

According to previous reports, pyrethroids are generally degraded via oxidation, hydrogenase and hydrolysis, and these microbial enzymes can usually convert toxic pyrethroids into small molecules that are less toxic or non-toxic (Demoute 1989). The sources of pyrethroid hydrolases are diverse; esterases which are part of the hydrolase enzyme family and have a critical role in degradation of pyrethroid, and breakage of the ester bond is generally considered to be the first step in the degradation of pyrethroids. Pyrethroid-degrading hydrolases have been reported in microbes, insects, plants, and animal cells (Bai et al. 2019; Wang et al. 2018; Yao et al. 2018). However, one study previously reported that either hydroxylases or dioxygenases may be accountable for the initial aerobic step in phenol degradation (Bhatt et al. 2020; Li et al. 2020). Oxygenase, an oxidoreductase, is important for the oxidation of polycyclic aromatic hydrocarbon compounds. It has been reported that bacteria degrade catechol primarily through *ortho-* and meta-pathways by catechol-1, 2-dioxygenase and -2, 3-dioxygenase, respectively (Tsai and Li 2007; Zhou et al. 2016). According to the genome-wide functional annotation of *C. freundii* CD-9, 25 hydrolases, 16 esterases, and 9 dioxygenase genes were manually identified. This represents a rich dioxygenase system and that may be one of the reasons why strain CD-9 can metabolize polychlorinated biphenyls, dioxins, and other aromatic compounds in a broad spectrum. However, different enzymes (carboxylesterase, oxygenase, and hydrolase) can be considered the catalyst of ester bond hydrolysis in the process of microbial degradation of pyrethroids.

**Fig. 5:**
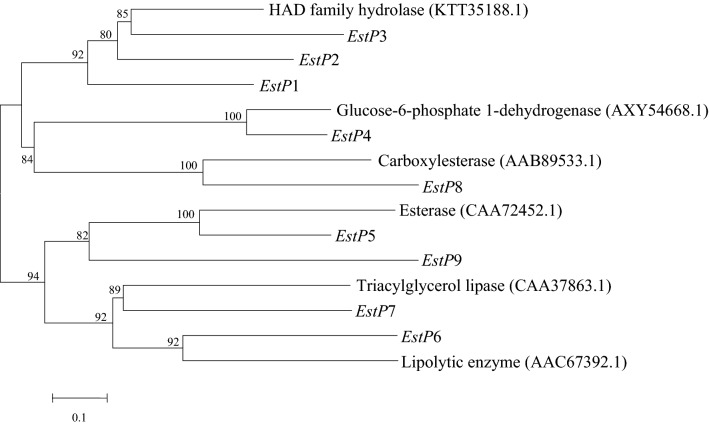
The evolutionary relationship between nine predicted proteins in the strain CD-9 genome and the reported proteins that degrade pyrethroids

The principal part of biodegradation mechanism of pyrethroids is the hydrolysis of their central ester bonds, which breaks down the original pesticide into carboxylic acids and alcohols (Saikia et al. 2005; Tallur et al. 2008; Wang et al. 2011; Xiao et al. 2015; Song et al. 2015; Pankaj et al. 2016). Subsequently, less toxic or non-toxic compounds are generated through further oxidation and dehydrogenation, finally entering the tricarboxylic acid cycle and mineralizing into H_2_O and CO_2_. After comparing the amino acid sequence of the reported pyrethroid-degrading enzyme with the gene information of the CD-9 genome, nine degrading enzymes with high homology were screened, as shown in Fig. 5. These are identified by locus tags PROKKA_00096, PROKKA_04415, PROKKA_05253, PROKKA_00708, PROKKA_00891, PROKKA_02977, PROKKA_04698, PROKKA_04561, and PROKKA_04927, and were subsequently named *estP*1 to *estP*9 respectively. In addition, four related degradation enzyme genes involved in the degradation of fenvalerate (PROKKA_00809, PROKKA_00814, PROKKA_02494, and PROKKA_04358), were identified and named *estP*10 to *estP*13.

### Gene expression analysis

**Fig. 6:**
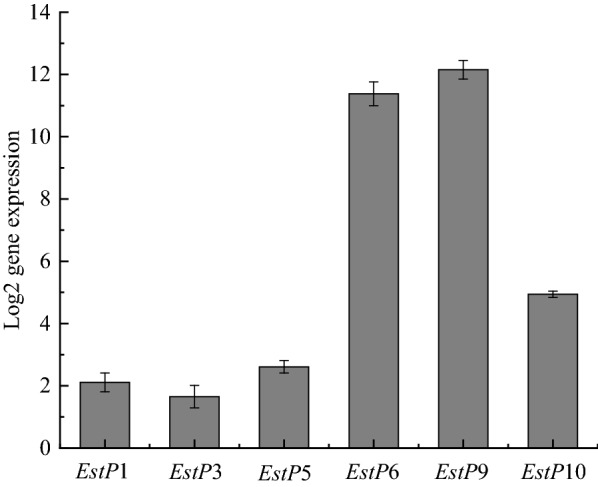
Relative expression of genes in *C. freundii* CD-9 under the presence of fenvalerate. All data are expressed as means ± standard deviation (SD)

According to the multistep degradation mechanism of pyrethroids, the chemical structure of fenvalerate, and the genomic sequences of *C. freundii* CD-9, it was speculated that there may be 13 genes involved in the degradation of fenvalerate. To verify this hypothesis and determine if the expression of these genes is dependent on fenvalerate availability, RT-qPCR was performed. The results showed that 6 of the 13 genes were significantly upregulated under the induction of fenvalerate by comparison with the untreated control group (Fig. 6). The change in *estP*9 was the highest (12-fold), followed by *estP*6 (11-fold). Phylogenetic analysis revealed that *estP*6 and *estP*9 from *C. freundii* CD-9 are likely to be lipolytic enzymes and esterases. The intermediate metabolites of pyrethroids are mainly diphenyl derivatives including 3-PBA (Zhan et al. 2018a, b). Studies have shown that the breakage of diphenyl ether bonds is the key link in the degradation process, and oxygenases play an important role in the breakage of diphenyl ether bonds, including dioxygenases and monooxygenases (Dashtban et al. 2009). Furthermore, we found that there is a special upregulated gene that belongs to gentisate 1, 2-dioxygenase related to the degradation of fenvalerate. These results confirm that the genes studied are related to fenvalerate degradation, and we speculate that these upregulated genes were strongly induced by fenvalerate.

## Discussion

The findings of this study suggest that *Citrobacter freundii* CD-9 can efficiently degrade not only fenvalerate but also other pyrethroid pesticides. The ease of pesticide degradation by microorganisms depends on the relative molecular mass of the pesticide, spatial configuration, and hydrophilicity of the substituents. Irrespective, microorganisms perform a significant role in the degradation of organic pollutants (Arbeli and Fuentes 2007). The degradation ability and sensitivity of strain CD-9 to the four pyrethroid pesticides differed, perhaps because of the different chemical structures and physicochemical properties of the different pyrethroid pesticides or the different enzyme genes activated in the bacteria.

**Fig. 7:**
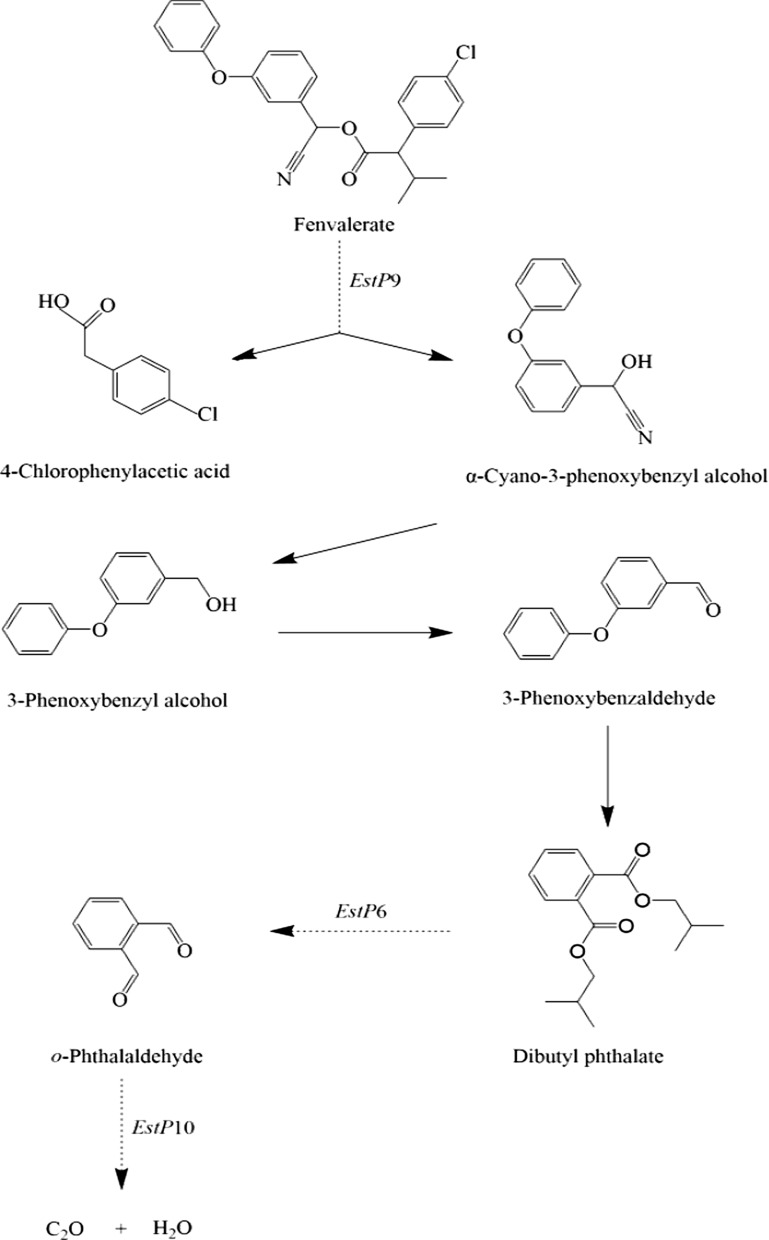
Proposed pathway of fenvalerate degradation by *C. freundii* CD-9

*Citrobacter freundii* CD-9 is a newly identified strain that can be used for bioremediation of agricultural crops and ecological environments contaminated by pyrethroid pesticides. To further investigate the possible mechanism of fenvalerate degradation, we conducted a genome-wide analysis of *C. freundii* CD-9 and found that this strain possesses an abundance of metabolic genes involved in diverse metabolic pathways and has a powerful potentiality to metabolize exogenous chemicals. Most notably, it contains several genes related to fenvalerate degradation, the most critical of which are esterase genes and some oxygenase genes. Based on the analysis of reported fenvalerate degradation products, genome-wide analysis, and RT-qPCR to verify the expression of relevant genes, the fenvalerate degradation pathway was preliminarily predicted, as shown in Fig. 7. During the degradation of fenvalerate, the cleavage of ester bonds caused by esterase (EstP9) is generally considered to be the first step, and 4-chlorophenylacetic acid and α-cyano-3-phenoxybenzyl alcohol are produced by the hydrolysis of ester bonds, which is in line with prior reports (Chen et al. 2011). Subsequently, owing to the structural instability of α-cyano-3-phenoxybenzyl alcohol, it was rapidly oxidized to 3-phenoxybenzaldehyde, followed by a complex series of reactions to produce dibutyl phthalate. Dibutyl phthalate is converted to *o*-phthalaldehyde by the action of the lipolytic enzyme EstP6. Subsequently, *o*-phthalaldehyde was transformed into catechol by gentisate 1, 2-dioxygenase (EstP10) then entered the tricarboxylic acid cycle to produce H_2_O and CO_2_, which was similar to earlier reports suggesting that gentisate oxygenase is involved in the metabolism of 3, 5-xylenol (Hoppe and Kemp 1980).

Up to now, there has been little research on the functional genes and enzymes involved in pyrethroid degradation. To further investigate the role of newly identified degradation genes, gene knockouts should be generated in future, and cloning and expression of each gene can open the door for the use of directed evolution to control the extent of pesticide degradation (Xu et al. 2020). With the progress of genetic engineering technology, it is possible to efficiently degrade fenvalerate by developing genetically engineered bacteria. Moreover, application models for environmental management of pollution from different food sources can be established through the development of degradation enzymes or bacterial formulations in the future, which are also the most effective ways to improve the biodegradation of pyrethroid pesticides. Hong et al. (2010), for example, introduced the methyl parathion gene *mpd* into *Sphingobium* sp. JQL4-5, a strain capable of degrading fenpropathrin, to successfully construct a strain capable of simultaneously degrading two pollutants. Overall, *C. freundii* CD-9 is a promising strain for use in pollutant degradation. This study provides a valuable reference for practical applications of pesticide biodegradation.

## Data Availability

The corresponding author is responsible for providing all the experimental data upon request.

## Abbreviations

HPLC: High-performance liquid chromatography
LB: Luria-Bertani
NR: Non-redundant
GO: Gene Ontology
COGs: Clusters of Orthologous Groups
KEGG: Kyoto Encyclopedia of Genes and Genomes
RT-qPCR: Reverse transcription polymerase chain reaction
